# “I use salt. However, I also use soy sauce, oyster sauce, sometimes chili sauce and….”: interviews with Australians of Chinese ancestry regarding reducing salt consumption for hypertension prevention

**DOI:** 10.1186/s12912-023-01576-3

**Published:** 2023-11-06

**Authors:** Alex Chan, Leigh Kinsman, Sally Wai-chi Chan

**Affiliations:** 1 School of Nursing and Midwifery, University of Newcastle, Newcastle, Australia; 2 School of Nursing, University of Wollongong, Wollongong, Australia; 3 La Trobe Rural Health School, La Trobe University, Bendigo, Australia; 4 Tung Wah College, Hong Kong SAR, China

**Keywords:** Health promotion, Hypertension, Chinese, Diasporas, Salt, Diet

## Abstract

**Background:**

High dietary salt consumption is a significant health issue in Chinese populations. This study identified the facilitators for and barriers to salt reduction for prevention of hypertension among Chinese Australians.

**Methods:**

An inductive qualitative study with semi-structured interviews (*n* = 8) was conducted with convenience samples recruited from social media. Adults who a) were over 18 years old, b) were of Chinese ancestry and c) had lived in Australia for at least 6 months were eligible for participation. Interview transcripts were transcribed and analysed using content analysis.

**Results:**

Four facilitators for and eight barriers to reducing salt consumption were synthesised from the narrative materials. The facilitators were: 1) individual perceptions of health benefits, 2) salt alternatives, 3) digital information and 4) increased awareness of negative health impacts from a high-salt diet. The barriers identified were: 1) negative physical changes not apparent, 2) inadequate salt-related health education, 3) hidden salt in food products, 4) inadequate food literacy, 5) pricing, 6) busy lifestyle, 7) low perceived susceptibility and 8) individual food taste preference and cooking habits. Peer and family influence had positive and negative effects on participants’ likelihood of reducing salt consumption.

**Conclusions:**

The facilitators for and barriers to maintaining a low-salt diet in Chinese Australians were multifaceted and interrelated. Future salt-reduction strategies should focus on the health benefits of reduced salt consumption and practical interventions such as salt alternatives and education on low-salt food choices and cooking methods and changing perceptions about salt reduction to become a social norm in the Chinese community.

**Supplementary Information:**

The online version contains supplementary material available at 10.1186/s12912-023-01576-3.

## Introduction

A high-salt diet is a modifiable lifestyle health issue and a major risk factor for hypertension [1]. The World Health Organization (WHO) recommends that adults consume no more than 5 grams (g) of salt per day [2], but high dietary salt consumption is common across the globe. For example, the average salt consumption in Australia was 9.6 g/day in 2018 [3], in Germany it was 8 to 10 g/day in 2008–2011 [4] and in China it was about 11 g/day in 2018 [5].

A recent systematic review [6] found that the amount of salt that was added during cooking or at the dining table contributed to more than half of the total daily salt intake in China, Costa Rica, Japan and India. This was significantly higher than for populations in some Western countries such as Australia, Canada and Denmark, where less than 25% of daily salt intake was acquired from discretionary sources and most salt intake came from processed foods [6]. Considering the adverse health outcomes of excessive salt consumption, migrants from high discretionary salt consumption countries, such as China, are at increased risk of combining traditional methods of adding salt during cooking with eating processed foods that are more available in western countries and can increase the incidence of hypertension. Although dietary practices often reflect individual food preferences, there are a range of factors influencing people’s dietary behaviours. These include parental food habits [7], cultural inheritance, social and peer support networks where people encourage each other to sustain healthy behaviours [8], and affordability of healthy foods [9].

Chinese are one of the biggest diasporic ethnic groups in the world [10], and hypertension is a major public health issue in China [11]. It was estimated in 2015 that 23.2% (*n* = 244.5 million) of Chinese adults had hypertension and 41.3% (*n* = 435.3 million) had pre-hypertension [11]. Chinese-born were the third largest group of overseas-born people living in Australia in 2021 [12]. Approximately 5% (1.2 million) of Australians were of Chinese ancestry (Chinese Australians) and the majority of these residents (75%) were first generation in the 2016 Census [13]. The design of the migration program deliberately attracts young and healthy people to the country so that they can contribute to economic and population growth. Therefore, new migrants tend to be healthier than native residents on arrival, but this healthy effect subsides over time [14]. A multinational study in 2018 asserted that the healthy migrant effect gradually subsided over 10 to 20 years in migrants to the USA, Australia and Canada [15]. However, little is known about the factors that influence Chinese migrants to manage their salt consumption in the host countries.

This qualitative study aimed to identify the facilitators for and barriers to reducing salt consumption for prevention of hypertension among Chinese Australians. To the best of our knowledge, a Canadian qualitative study of salt consumption behaviours among older Chinese migrants (mean = 60.8 years, SD = 11.7) with stage one hypertension is the only other published qualitative exploration of this topic [16]. No study has been published from the perspective of using salt reduction as a preventive strategy for hypertension in Chinese Australians. This study sought to identity the facilitators for and barriers to reducing salt consumption for prevention of hypertension in Chinese Australians. The study findings should inform the design of future salt-restriction strategies for this population group.

## Methods

### Design

This study was a qualitative descriptive study conducted as part of a larger mixed-methods study into dietary salt intake in Chinese Australians. This study was conducted during the Coronavirus pandemic in 2021 when human movement was restricted. Therefore, the phone interview approach was the most appropriate data collection method at that time. Moreover, phone interviews could overcome the geographical distance barriers and therefore the study could recruit individuals from different parts of Australia. The interviews were conducted in plain English or Chinese language (avoiding medical and chemical terms) so the participants could gain a better understanding of the questions and respond from their perspectives.

### Sample and setting

A convenience sampling method was used in this study. Participants were recruited from social media between February and May 2021. Recruitment materials with the first author’s email and phone number were posted in a range of Chinese Australian online groups and blogs including the Chinese community in Australia, Chinese business, and LGBTIQ + (lesbian, gay, bisexual, transgender, intersex, queer or questioning, or another diverse gender identity) groups on Facebook, Instagram, WeChat and Weibo. The participants’ information sheet and consent form (English only) were sent to the interested individuals’ nominated email addresses for their consideration. If the individuals wished to participate and returned the signed consent form, phone interviews were scheduled at their convenience.

Prior to the beginning of each interview, the first author informed the participants that participation was voluntary, confirmed their understanding of the study, provided an opportunity for them to ask questions, advised their right to withdraw from the study at any time, and that they could choose whether the interview was audio-recorded. The notetaking and audio-recording procedures were explained at the beginning of each interview, and participants were reminded not to provide personal identifiable information during the recording.

The study inclusion criteria were a) over 18 years old, b) of Chinese ancestry, and c) had lived in Australia for at least 6 months. Adults who were unable to communicate verbally in Chinese or English, or complete a written consent form in English were excluded from the study. This study aimed to only recruit bilingual Chinese Australians who were able to find, understand, and use the salt-related health information that was designed for the general Australian population. Therefore, this study could identify the unmet needs of the Chinese Australians.

The methodological principle for determining the sample size in this phone interview study was that of data saturation. Recruitment and data collection continued until data saturation as reached, that is, new data repeated what had already been raised in previous data [17].

### Data collection

This study consisted of a formative stage and a process stage. Data were collected using semi-structured phone interviews. A set of guiding questions was developed based on the findings from a quantitative study on this topic conducted by the authors (AC, SC and LK) [18] and a literature review [19]. The interviews were begun with a general health question, ‘Do you have high blood pressure or kidney disease?’ and then followed by a series of questions designed and based on the Health Belief Model (HBM) [20] that covered the salt-related health knowledge, perceptions of health risks associated with a high salt diet, perceived health benefits of a low salt diet and barriers to follow the recommended salt reduction strategies. Depending on the participants’ responses, questions used in the interviews included ‘What do you think about the health benefits of reducing the current level of salt intake to you and your family?’, ‘Can you identify 3 barriers that prevent you from following a low-salt diet?’, and ‘Can you tell me the reasons why you perceive that they are your barriers? Can you share your experience with me?’ (see Additional file 1). Interviews were conducted by the first author to ensure consistency and were conducted in English or Chinese, depending on participants’ preferences.

A pilot study (*n* = 1) was conducted in the formative stage. The participant was recruited using the same inclusion criteria through the first author’s social network. The pilot study aimed to test the guiding questions as well as identify and fix any possible technical issues with the interview prior to data collection (process stage). The findings from the pilot study were not included in the data analysis.

Interviews were conducted at times of the participants’ convenience and lasted between 30 and 60 min. Interview records were fully transcribed using an automated transcription software program. Interviews conducted in Chinese were initially transcribed to text and then translated to English by the automated transcription software. The accuracy of transcriptions was confirmed line-by-line by the first author.

### Data analysis

**Fig. 1:**
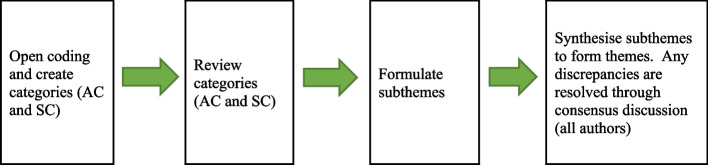
Flow diagram of the coding and review process

An inductive content analysis method was used to analyse the data [21]. Each transcription was coded individually by two authors (AC and SC). The process included open coding, creating categories and subthemes, and formulating abstractions (themes) (Fig. 1). First, each category was named using the content words from the collected data. Second, categories were reviewed individually by the authors (AC and SC). Third, subthemes were formed by grouping similar or related categories together. The purpose of creating subthemes was to increase insight into the phenomenon by comparing and condensing the categories [21]. Subthemes were further collated and synthesised to form abstractions (themes). Any discrepancies were resolved through consensus discussion. A descriptive analysis method was used to analyse the demographics of the participants.

### Rigour

Rigour and trustworthiness were maintained by following the criteria of rigour, which include credibility, applicability, auditability and confirmability, as described by Sandelowski [22]. Credibility was ensured as all interviews were conducted by one author and data were collected in the same style. Data analysis was completed by two authors independently. Discrepancies were reviewed and discussed until agreement was reached. The applicability of this study was warranted by recruiting participants from different types of social media networks, so that participants were not from one particular age group, or social or cultural background. Interviews were audio-recorded with the consent of participants and then transcribed verbatim. This process not only promoted auditability, but also allows readers to assess the transferability and applicability of the findings. To promote confirmability, participants’ responses were quoted in the results section, allowing readers to evaluate the quality of the interpretation of the authors as well as the authenticity. The Standards for Reporting Qualitative Research checklist was used to guide the preparation of this report [23].

### Ethical considerations

Ethics approval was granted by the Human Research Ethics Committee at the University of Newcastle, Australia (approval number: H-2019–0180). Participants were offered copies of their interview transcripts. The first author confirmed participants’ understanding of the study and consent before the beginning of the interviews. In appreciation of their time to participate in the telephone interviews, participants were given $50 gift vouchers.

## Results

### Description of participants

After the sixth interview (excluding the pilot interview), no new themes emerged that could be added to the overall story. Two additional interviews were conducted to ensure that there were no further new emergent themes. For the eight phone interviews conducted, four participants were men and four were women. The participants’ ages were evenly distributed across four age groups, ranging from 18 to 24, 25 to 34, 35 to 44 and 55 to 64 years old. Although the age range was wide, responses to the guiding questions were similar, especially when discussing barriers to salt reduction. All participants had tertiary education qualifications. Two participants had annual incomes around or above the national average annual income in Australia of AU$89,003 (US$61,348) [24]. All participants reported no history of hypertension or kidney disease and no formal salt-related health information was received in Australia. Some participants mentioned that they had received the information in their home country. Further details about the salt-related health information and education will be provided in Section Inadequate salt-related health education. The participants’ demographic data are presented in Table 1.

**Table 1:** Demographic characteristics of the participants

		***N* = 8 (%)**
Gender	Men	4 (50.0)
	Women	4 (50.0)
Age group	18–24	2 (25.0)
	25–34	3 (37.5)
	35–44	2 (25.0)
	45–54	0 (0)
	55–64	1 (12.5)
Place of birth	Australia	2 (25.0)
	Hong Kong	4 (50.0)
	Taiwan	1 (12.5)
	China (Shandong)	1 (12.5)
Marital status	Single	5 (62.5)
	Married	2 (25.0)
	Living with partner	1 (12.5)
Educational level	College/Bachelor's degree	3 (37.5)
	Master's degree or higher	5 (62.5)
Average weekly income	< $399	4 (50.0)
	$800-$1249	1 (12.5)
	$1250-$1900	1 (12.5)
	> $2000	1 (12.5)
	No response	1 (12.5)
Occupation	Office worker	1 (12.5)
	Professional	1 (12.5)
	Student	3 (37.5)
	Other—media, caterer	2 (25.0)
	Unemployed	1 (12.5)
Location	New South Wales	5 (62.5)
	Queensland	2 (25.0)
	Tasmania	1 (12.5)
Length of residence in Australia	1–5 years	3 (37.5)
	5–10 years	1 (12.5)
	Over 10 years	2 (25.0)
	Born in Australia	2 (25.0)

### Content analysis

A total of four themes and eleven subthemes emerged from the narrative materials (Table 2). Four themes related to the facilitators for and barriers to reducing salt consumption were: 1) perceived health benefits; 2) resources and support; 3) wide availability of commercial food products; and 4) factors affecting health behavioural change. The details of the themes, subthemes and representative quotes are presented in Table 3.

**Table 2:** Facilitators for and barriers to reducing dietary salt consumption

Theme	Facilitators	Barriers
**Perceived health benefits**	• Individual perceptions	• Physical changes (benefits) are not apparent
	• Negative health impacts	
**Resources and support**	• Salt alternatives	• Inadequate salt-related health education
	• Digital information	
**Wide availability of commercial food products**		• Hidden salt in commercial food products
		• Inadequate food literacy (food labelling)
		• Affordability and convenience
**Factors affecting health behavioural change**	• Peer influence^a^ • Family influence^a^	• Individual food taste and cooking habits • Low perceived susceptibility • Peer influence^a^ • Family influence^a^

^a^Both a barrier and a facilitator

**Table 3:** Themes and subthemes for facilitators for and barriers to reducing dietary salt consumption

Theme	Sub-theme	Representative quote
**Facilitators**		
**Perceived health benefits**	Individual perceptions	1) “… I ate heavily flavoured foods in the past. Because my skin condition became poor [unwell], then I changed to eat light-flavoured foods. Obviously, my skin condition improved, and I am more used to eating light-flavoured foods. I think it is good, because heavy-flavoured food does not mean the taste is better. Simple food tastes good. I feel better physically and mentally, not that easy to get tired when I started the light-flavoured diet.” (P2) 2) “I found that I felt very uncomfortable, had indigestion or very tired after I eat too much food with MSG or strong taste.” (P4)
	Negative health impacts	3) “I expect some intimidating information, let people see the consequences of salt… Or the effects of excessive salt intake on cardiovascular system, will it block the cardiovascular system? It is good to have a picture showing a blood vessel narrowed down to a line.” (P5) 4) “I think it's better to have some harmful side [information] about eating too much salt… the best option is let them [people] know that how bad if they take too much salt …” (P7)
**Resources and support**	Salt alternatives	5) “The restaurant foods are delicious because they put a lot of salt and seasonings. So homemade meals taste relatively light. If there is a way to make the food taste good without too much salt, then I’d use less salt…I cook for myself; I try to use different herbs instead. Taste good but not too salty.” (P4) 6) “Use vinegar, black pepper instead of salt to enhance the food flavouring.” (P3) 7) “…you can provide some guidance, such as low added salt and condiment recipes. The biggest problem with Chinese cooking is using plenty of condiments. The salt content in condiments is unclear… I do not know the amount of salt [in the paste/sauces]. I don't know whether it has excessive amount of salt.” (P5)
	Digital information	8) “Soft copy is easier for me because, you know, my parents are around 60 years old. The info [information] is not only useful for me. If I got such information, I will share it via WeChat with my parents too.” (P7) 9) “Hard copy information maybe you look just for once and then you throw it away. So, like for me, it's better to have digital information. I can put on my computer or somewhere else.” (P8)
**Barriers**		
**Perceived health benefits**	Physical changes (benefits) are not apparent	10) “Stop eating salt, I won't lose weight. What is the positive reinforcement? A low-salt diet will not improve the skin condition. Stop eating sugar and oil, the body weight is reduced.” (P5)
**Resources and support**	Inadequate salt-related health education	11) “Yes, not much advertising about salt reduction in Australia. Haven't received any [education] in Australia… I don't think the public has a strong idea of salt reduction as [much as] sugar reduction.” (P1) 12) “My knowledge about the health impacts of salt is just from some random information online and they keep emphasizing that a lot of people currently, taking too much salt… I still believe if I can reduce the amount of salt that I take daily, that should be better for my health.” (P7) 13) “Local GP focuses on secondary prevention. Salt consumption is not a topic that the GP would bring up for discussion.” (P3) 14) “People don’t know the health risk associated with high salt intake. People know that a high sugary diet is a risk for diabetes. A high-salt diet is known to be a cause of hypertension, but hypertension is not perceived as a significant health issue… High blood pressure is not serious enough to die.” (P3)
**Wide availability of commercial food products**	Hidden salt in food products	15) “I think Asian food may need a lot of different seasonings. There is a lot of salt in different seasonings. Therefore, the food tastes good… For example, stewed chicken with potatoes may contain oyster sauce, and light and dark coloured soy sauces. It is difficult to calculate the amount of salt in these three condiments…” (P2) 16) “I believe that my daily food contains salt, and I am not aware. For example, processed meat, bacon and ham, they have salt. Even if a slice of bread has salt… A lot of opportunities to buy processed meat. And there's a lot of ingredients inside the processed meat that I don't know.” (P5) 17) “The processed food manufacturers, they add salt [to the food products] that it's also a barrier… I would like them [processed foods] to have less salt but it doesn't seem to be a change [over time].” (P6)
	Inadequate food literacy (food labelling)	18) “It is difficult to calculate the amount of salt in the condiments… And each brand has different food labels, and the salt content is not the same, so I won't check each time, too much trouble…” (P2) 19) “I don't really know the ingredients. Even they have the ingredients label on the back, but they're sort of medical [chemical] terminology. I don't understand what they are.” (P7)
	Affordability and convenience	20) “Foods with high salt content are often cheaper in supermarkets. So, I buy them because the price is lower.” (P4) 21) “If I really want to make homemade sauce, I will make my own chicken broth when I'm free. Then I can be sure that the broth does not contain salt… But I'm not free to make chicken broth every day, so sometimes I need chicken extract.” (P5)
**Factors affecting health behavioural change**	Individual food taste and cooking habits	22) “Adding a pinch of salt” (P1) 23) “Salt is usually added to the meals last. I try the taste first before I add salt to the meals… when I add a condiment whether is soy sauce or other seasoning to the meals, I don't check the amount of salt that is being added to the meals.” (P2) 24) “I rely on my experience. The dish that I have never cooked, I will try the taste at first. But if I've already cooked the dish before, I add salt to the dish without tasting it.” (P5) 25) “It's sort of depends on my taste…, I sort of tweak that, tweak it [salt] according to my taste… I add a little bit more, little bit more at time for the individual meal.” (P6) 26) “I cook in bulk, so I make a lot at once… I use salt. However, I also use soy sauce, oyster sauce, sometimes chili sauce and yeah, that is quite normal… I used to measure but now I am lazy. Just kind of that should be one spoon. So, myself, I don't measure, but I think I could I have the good sense of how much I put in without that sort of measures or something.” (P7)
	Low perceived susceptibility	27) “I don't exactly have heart issues at the moment. So, I know I don't have as much incentive as someone who has heart issues… if I actually have heart issues, then I would probably try a bit harder. But I don't [have heart issues].” (P6)
**Barriers and Facilitators**		
**Factors affecting health behavioural change**	Peer influence	28) “The barrier is eating out with friends. I do not want others to feel uncomfortable. I won't ask my friends to follow a low salt and sugar diet. If I invite my friends home for dinner, I won't request my friends to eat light-flavoured foods because of my dietary preferences.” (P2) 29) “Eating out is relatively difficult, because gathering with friends and share foods… (P4) 30) “People who I live with enjoy eating highly flavoured foods. I would add extra sugar and salt in cooking.” (P4) 31) “Eating with friends is harder, because I can't just think about myself**…**” (P5) 32) “I lived with a roommate… she told me if she ate too much salt, she had abdominal pain and discomfort. I realised I need to reduce my salt and sugar intake because of her.” (P2)
	Family influence	33) “Family members [parents] instructed not to eat too much salty foods. My family's dietary habits recommend not to eat pickled foods or salty foods. My mother is a renal nurse, so we eat salt as little as possible… My family does not allow us to eat salted fish, fried rice with salted black beans and canned cooked meat products. All canned processed foods are basically not allowed.” (P1) 34) “I cannot be too strict [to my family members]. I have to relax sometimes. If forcing them to eat a low-salt diet every day, it is easy to cause family conflicts.” (P5) 35) “I think it is easier because in Australia. I'm single, so I cook for myself. I can control the flavour in my food…” (P8)

### Perceived health benefits

#### Individual perceptions of health benefits from their previous experience with following a low salt diet

Individuals’ perceptions of immediate health benefits from lowering their salt consumption may facilitate a health behavioural change: “I found that I felt very uncomfortable, had indigestion or very tired after I eat too much food with MSG [monosodium glutamate] or strong taste.” (P4). Further, participants with chronic medical conditions might have experienced some psychological or physical effects (benefits) when reducing their salt consumption. This might have reinforced their perceptions of the benefit from changing their salt-related behaviours: “… I ate heavily flavoured foods in the past. Because my skin condition became poor [unwell], then I changed to eat light-flavoured foods. Obviously, my skin condition improved, and I am more used to eating light-flavoured foods. I think it is good, because heavy-flavoured food does not mean the taste is better. Simple food tastes good. I feel better physically and mentally, not that easy to get tired when I started the light-flavoured diet.” (P2). In summary, individuals’ experience with the effects of reducing dietary salt consumption could have a significant influence on their perceptions of health benefits from sustaining salt-related health behavioural changes.

#### Negative health impacts

A good understanding or awareness of the negative health impacts associated with a high-salt diet may promote salt-related behavioural changes. Participants reported that they would like to know more about the negative impacts of a high-salt diet on their health: “I think it's better to have some harmful side [information] about eating too much salt…, the best option is let them [people] know that how bad if they take too much salt …” (P7). Moreover, health education with infographics to illustrate the health problems associated with a high-salt diet may help people to understand the health benefits of reducing their overall salt consumption: “I expect some intimidating information, let people see the consequences of salt… Or the effects of excessive salt intake on cardiovascular system, will it block the cardiovascular system? It is good to have a picture showing a blood vessel narrowed down to a line.” (P5).

#### Physical changes (benefits) are not apparent

Most of the participants did not perceive that a low-salt diet had a positive effect on their health because the results of salt reduction were not obvious: “Stop eating salt, I won't lose weight. What is the positive reinforcement? A low-salt diet will not improve the skin condition. Stop eating sugar and oil, the body weight is reduced.” (P5).

### Resources and support

#### Salt alternatives

Food taste is a priority for many people and is considered important for a sensation of well-being. Participants (*n* = 8) noted that the only strategy to avoid hidden salt in processed and restaurant food was through careful food selection and cooking with alternative flavourings at home. For example, “The restaurant foods are delicious because they put a lot of salt and seasonings. So homemade meals taste relatively light. If there is a way to make the food taste good without too much salt, then I’d use less salt…I cook for yourself; I try to use different herbs instead. Taste good but not too salty.” (P4). In general, using “vinegar, black pepper instead of salt to enhance the food flavouring” (P3) would be a more sustainable option than consuming foods with little or no flavour for the people who perceived food taste as a priority.

In Chinese cooking, salt and condiments are often added during the cooking process. One of the challenges for Chinese people is that salt-rich condiments such as soy sauce for cooking are readily available and commonly used. Participants noted that they would appreciate some culturally tailored information about food choices and preparation methods: “… you can provide some guidance, such as low added salt and condiment recipes. The biggest problem with Chinese cooking is using plenty of condiments. The salt content in condiments is unclear… I do not know the amount of salt [in the paste/sauces]. I don't know whether it has excessive amount of salt.” (P5).

#### Digital information

Many participants preferred to receive salt-related health information in digital format: “Soft copy is easier for me because, you know, my parents are around 60 years old. The info [information] is not only useful for me. If I got such information, I will share it via WeChat with my parents too.” (P7). In addition, electronic materials could be stored on their computers for future reference: “Hard copy information, maybe you look just for once and then you throw it away. So, like for me, it's better to have digital information. I can put on my computer or somewhere else.” (P8).

#### Inadequate salt-related health education

Excessive salt consumption is not perceived as important for one’s health. Very few participants (*n* = 2) knew the WHO’s recommended daily salt intake (5g of salt/day or 2000mg of sodium/day). The current salt-related health education about the complications of excessive salt consumption was not well-received or sufficient to raise participants’ awareness of the need to reduce salt consumption to reduce the risks of certain medical conditions such as hypertension: “People don’t know the health risk associated with high salt intake. People know that a high sugary diet is a risk for diabetes. A high-salt diet is known to be a cause of hypertension, but hypertension is not perceived as a significant health issue… High blood pressure is not serious enough to die.” (P3).

Participants reported a lack of formal salt-related education, especially on the topic that negative health issues associated with excessive salt intake may reduce the effects of promoting a healthy lifestyle through dietary modifications and interventions. One participant noted: “My knowledge about the health impacts of salt is just from some random information online and they keep emphasising that a lot of people currently, taking too much salt…” (P7). Overall, participants in this study indicated that there was room for improvement in salt-reduction health campaigns: “Yes, not much advertising about salt reduction in Australia. Haven't received any [education] in Australia…I don't think the public has a strong idea of salt reduction as [much as] sugar reduction.” (P1). On another note, “Local GP [general practitioners] focuses on secondary prevention. Salt consumption is not a topic that the GP would bring up for discussion.” (P3).

### Wide availability of commercial food products

#### Hidden salt in food products

Hidden salt in food is a public health concern. Commercial foods (including the sauces and condiments that Chinese people commonly use in cooking), restaurant food, fast food, and processed food are great challenges for people who want to reduce their salt consumption. Commercial foods often contain a variety of seasonings, particularly salt, which is commonly added to the foods for flavouring and for a preservative purpose [25]. Although commercial foods contain a high level of salt, it is used for convenience and taste. People may ingest excessive amounts of salt without being aware: “I believe that my daily food contains salt, and I am not aware. For example, processed meat, bacon and ham, they have salt. Even if a slice of bread has salt… A lot of opportunities to buy processed meat. And there's a lot of ingredients inside the processed meat that I don't know.” (P5). Consuming cultural foods may also increase the ingestion of hidden salt: “I think Asian food may need a lot of different seasonings. There is a lot of salt in different seasonings. Therefore, the food tastes good… For example, stewed chicken with potatoes may contain oyster sauce, and light and dark coloured soy sauces. It is difficult to calculate the amount of salt in these three condiments…” (P2). On the other hand, inadequate food reformulation regulations and market demand reduce low-salt food product availability: “The processed food manufacturers, they add salt [to the food products]. That's also a barrier… I would like them [processed foods] to have less salt but it doesn't seem to be a change [over time]…” (P6).

#### Inadequate food literacy

Food labels are not often written in plain language and use non-standard units of measurement, creating difficulties for consumers in comprehending them and informing their decisions about restricting salt intake from commercial food products. A participant reported: “I don't really know the ingredients. Even they have the ingredients label on the back, but they're sort of medical [chemical] terminology. I don't understand what they are.” (P7). This makes it more difficult to limit salt consumption and discourages people from monitoring their salt consumption: “It is difficult to calculate the amount of salt in the condiments… And each brand has different food labels, and the salt content is not the same, so I won't check each time, too much trouble…” (P2).

#### Affordability and convenience

Processed foods are widely accessible, taste good and are at affordable prices. One participant said: “Foods with high salt content are often cheaper in supermarkets. So, I buy them because the price is lower.” (P4). In some cases, participants (*n* = 2) were aware of the hidden salt issue, but it was difficult to avoid commercial foods because of their busy lifestyles and personal choices, such as the taste, or a lack of desire to cook. A participant noted that the only strategy to avoid hidden salt in foods was to cook from scratch at home, but this could be a challenge to the modern lifestyle: “If I really want to make homemade sauce, I will make my own chicken broth when I'm free. Then I can be sure that the broth does not contain salt… But I'm not free to make chicken broth every day, so sometimes I need chicken extract.” (P5).

### Factors affecting health behavioural change

#### Individual food taste and cooking habits

The usage of salt is not always based on accurate quantity measurement. None of the participants in this study reported they measured their salt usage. Their behaviour regarding dietary salt consumption is mostly driven by their food taste preference and cooking habits such as “adding a pinch of salt” (P1); or “it's sort of depends on my taste… I sort of tweak that, tweak it [salt] according to my taste… I add a little bit more, little bit more at the time for the individual meal.” (P6). One participant said: “Salt is usually added to the meals last. I try the taste first before I add salt to the meals… when I add a condiment, whether is soy sauce or other seasoning to the meals, I don't check the amount of salt that is being added to the meals.” (P2). Another participant reported: “I rely on my experience. The dish that I have never cooked, I will try the taste at first. But if I've already cooked the dish before, I add salt to the dish without tasting it.” (P5).

Some people may cook in bulk so they do not have to cook every day. This practice may suit an individual’s lifestyle and work schedule. However, this presents a challenge when monitoring and measuring daily salt consumption as salt intake is dependent on serving size and other foods consumed during the day: “I cook in bulk, so I make a lot at once… I use salt. However, I also use soy sauce, oyster sauce, sometimes chili sauce and yeah, that is quite normal…” (P7).

#### Low perceived susceptibility

In some participants, the long-term health benefits of salt reduction are undervalued because of young age and/or current health status. This results in a low perceived susceptibility to the health impacts of a high salt diet, “I don't exactly have heart issues at the moment. So, I know I don't have as much incentive as someone who has heart issues… if I actually had heart issues, then I would probably try a bit harder. But I don't [have heart issues].” (P6).

#### Family and peer influence

Peers may influence people’s dietary habits positively or negatively. Consumption of food is part of the social fabric through eating with peers and family members. A strict low-salt diet may disappoint peers when eating together and also may create family conflicts. Participants reported that they compromise on their dietary practice and cook according to their friends’ food taste preferences: “People who I live with enjoy eating highly flavoured foods. I would add extra sugar and salt in cooking.” (P4); and “I lived with a roommate… she told me if she ate too much salt, she had abdominal pain and discomfort. I realised I need to reduce my salt and sugar intake because of her.” (P2).

Dining out was a great challenge regarding salt reduction for many participants (*n* = 4) because the food was commercially prepared, and often contained a mix of additives such as sauces and condiments to make the food more appealing and tastier. It is important to note that participants worry about disappointing their friends if they order bland foods that have little or no flavour if they share their meals. Participants reported: “Eating out is relatively difficult, because gathering with friends and share foods.” (P4); and “Eating with friends is harder, because I can't just think about myself…” (P5). The fundamental factor is that a meal needs to be tasty so that friends can enjoy the meal together.

Chinese family culture, customs and food practices increase the difficulties for family members who follow a low-salt diet. Participants found that it would be a challenge when other family members refuse to follow a low-salt diet. They would add extra seasonings to flavour the foods to avoid disappointing their family members and family conflicts: “I cannot be too strict [to my family members]. I have to relax sometimes. If forcing them to eat a low-salt diet every day, it is easy to cause family conflicts.” (P5). In some cases, the parents can be a positive role model in following a low-salt diet and make a lifelong impression on their offspring: “My mother is a renal nurse, so we eat salt as little as possible… My family does not allow us to eat salted fish, fried rice with salted black beans and canned cooked meat products. All canned processed foods are basically not allowed.” (P1).

## Discussion

This is the first known qualitative study that has identified the facilitators for and barriers to reducing dietary salt consumption among Chinese Australians. It is also the first study of its kind on adults of Chinese ancestry from the perspective of using salt reduction for primary prevention of hypertension in Western countries. Excessive dietary salt consumption is a significant risk factor for hypertension; it increases morbidity and mortality from cardiovascular disease [26]. Culturally appropriate and relevant preventive measures for hypertension are important for minority ethnic groups such as overseas Chinese people who reside in a culture where Chinese cultural practices are used by a minority, as in Western countries. Compared with the Chinese Canadian study (with mean age of 60.8 years, SD = 11.7) in 2019 [16], our study successfully recruited participants from younger age groups, who were mostly single and without a history of hypertension. The study findings could be used to tailor current preventive strategies, in particular, primary interventions appropriate to the needs of Chinese Australians. Moreover, the findings provide preliminary evidence to assist in the design of salt-reduction strategies for overseas Chinese people around the globe.

The identified themes related to the facilitators for reducing salt consumption in Chinese people showed that appropriate salt-related education such as increased awareness of low-salt food choices is the most effective strategy to reduce a person’s overall salt consumption. People who prioritise food taste over their health may appreciate the use of alternative options to salt to make food tasty. A recent study reported that almost 80% of daily salt consumption in China was from homemade foods [27]. Adding salt and salty condiments during cooking is also a common practice among people in Benin, Guinea, Kenya, Mozambique, and Seychelles [28]. A Japanese study in 2018 reported that monitoring and controlling the use of salt in homemade foods was an effective strategy for reducing salt consumption [29]. In general, all participants agreed that cooking at home was the most practical way to minimise their salt consumption because salt usage was under their control. Therefore, reducing the consumption of discretionary salt is an important area to target in salt-reduction education both at the individual and household levels.

In this study, two participants recommended the inclusion of infographics in salt-related health education so that individuals can visualise the health consequences of a high-salt diet. The use of damaged organ images in health education has proven to be an effective strategy to promote and sustain health-related behavioural changes such as smoking cessation, and may meet the identified facilitator of being seen to be of immediate health benefit [30]. According to the HBM, an improvement in the perception of health benefits from reducing salt consumption could sustain behavioural change [20]. Moreover, infographics in digital format may further increase the impact of the content. Evidence shows that digital health infographics not only improve the knowledge transfer process from health care providers to the target consumers, but also assists consumers to retain the information [31]. Further, health consumers can share the infographics on their online social networks, resulting in dissemination of the health messages to the broader community [31]. It is important to acknowledge that participants in this study were recruited from social media. Therefore, they were likely to have a higher level of digital literacy skill than other individuals from the same cultural background. However, the cultural relevance of the content and their health literacy skills may affect their ability to appraise and use online health information to make informed health decisions [32]. Therefore, health care providers should invite the relevant stakeholders to codesign digital salt-related education for this group of the population.

The barriers to reducing salt consumption identified in this study were mostly associated with the inadequacy of culturally relevant salt-related health education, commercial food products, social and family influences, low perceived susceptibility and personal food taste and cooking habits. As mentioned previously, salt and a variety of salty condiments are commonly added during food preparation to enhance the appeal and taste of food. Participants in this study noted that they were aware of the possibility of high-salt content in restaurant foods and fast foods, but they were unable to minimise their salt consumption when dining out. This was because the ingredients in restaurant foods were not known, and restaurants had no legal obligation to provide such information in their menus. This finding is consistent with a recent literature review that found lack of availability of low-salt food on menus was a barrier to salt reduction when dining out [33].

Apart from cultural practices, humans’ health behaviour may also be influenced by their peers, community, and society [7]. In this study, our participants reported negative influences from their peers, such as unspoken peer pressure to avoid ordering bland dishes when dining out with friends. This is likely related to social norms and culture within the Chinese community and the wider society. Unlike sugary food, a well-known health risk factor for diabetes [34] and obesity [35], salt reduction is a relatively less openly discussed health topic among peers. It is important to note that although hypertension is the most common complication of a high-salt diet, hypertension is considered to be a less life-threatening condition (P3). This misperception about hypertension may result in individuals’ exercising inadequate self-care practices to prevent hypertension. As stated by the HBM, if individuals do not perceive that the condition is a threat to their overall health, it is very unlikely that they will follow the recommended interventions and sustain a health-related behavioural change, especially individuals with low perceived susceptibility [20]. This indicates that there is room for improvement in salt-related health education for this population group. The design of future health education should not only place more emphasis on culinary traditions, but also on the practical strategies that are relevant and appropriate for Chinese Australian dietary customs.

Despite food labelling being required for all processed foods, most food labels fail to deliver the necessary health-related information for people to make healthier food choices. The comprehension of food labels often requires high-level literacy and numeracy skills [36], and the utilisation of information on food labels may further be impaired by the chemical codes and non-standard units of measurements on the labels. A study in Hong Kong reported that 48% of the participants in their study had difficulties in determining serving and portion sizes [37]. There is evidence that people may develop their own strategies for salt reduction or improve the level of adoption of the recommended salt-restricted interventions if appropriate resources and support are available [38]. Thus, low-salt recipes and salt substitutions are essential parts of salt-related education [39]. People need to know the healthier options to use, instead of just giving up their habitual food consumption altogether. A couple of participants in this study reported that their salt usage in cooking was determined by their food taste preferences, which is one of the key determinants of salt use identified by the participants. Therefore, knowing alternatives to salt, such as herbs and potassium-enriched salt substitutes, is an important motivational factor. People can reduce their salt consumption but do not have to skimp on what they perceive as flavour.

An absence of convenient and reliable methods of salt calculation and ignorance of the recommended daily salt intake are significant barriers to adhering to the recommended salt intake of 5g per day. The study found that most participants (*n* = 6) did not know the WHO’s recommended daily salt intake. In other words, many participants did not know how much salt is too much and they might not fully understand the importance of salt reduction. Not knowing the recommended amount of daily salt consumption would result in under-recognising their salt consumption and its impacts on health. Three participants reported that the current salt-related health education was inadequate to highlight the health risks caused by excessive salt consumption. This finding suggests that knowledge about the health risks associated with excessive dietary salt consumption has room for improvement.

Prior to this study, evidence regarding the facilitators for and barriers to reducing salt consumption among people of Chinese ancestry in Australia or elsewhere was very limited, particularly where Chinese cultural dietary practices are employed by a minority, given that reducing salt consumption not only reduces the risks for hypertension, but also the risk of other chronic health conditions linked to hypertension, such as stroke and kidney disease. The Chinese Australian community and perhaps the Australian general public should be made aware of the health benefits of a low-salt diet and encouraged to reduce their salt consumption as a cost-effective strategy for primary prevention of hypertension. The research findings could inform the practice of current preventive strategies for dietary salt-related hypertension for individuals from a Chinese background living in Western countries.

In summary, based on the findings of this study, current salt reduction strategies for the Chinese Australian population group should place more emphasis on the health benefits of reducing salt consumption. More importantly, the strategies must be appropriate to their unique dietary preferences. The strategies would be more feasible and acceptable if they could assist Chinese Australians to explore culturally relevant low-salt options that do not compromise too much on taste. In addition, study participants reported that digital infographics were the most preferable format for health education. In this case, graphic visual representations of information may make a positive impact on people’s perceptions about reducing salt consumption and inspire the target audiences into action for the prevention of hypertension.

### Limitations

This study has some limitations. First, the study used a convenience sample, and the participants were recruited from social media. As a result, the participants may be more digitally literate than other individuals from the same cultural background. People who are digitally literate are generally more confident in searching for information online. Also, the participants needed to be able to give informed consent in English, had tertiary education quantifications and were relatively young. Therefore, the findings may only represent a group of individuals of Chinese descent in Australia who are well-educated and digitally literate, and have a special interest in the available health information online. This may restrict the transferability of the findings to Australians of Chinese ancestry outside the study sample. Second, the participants might have provided responses that they thought were appropriate in the interviews. Therefore, social desirability bias may exist in this study.

## Conclusion

Participants in this research study identified various factors influencing the salt-related behaviours of Chinese Australians. Our findings showed that: 1) individual perceptions of health benefits, 2) availability of salt alternatives, 3) digital information and 4) increased awareness of negative health impacts from a high-salt diet are the main facilitators for salt-related behavioural changes. The identified barriers to reducing dietary salt consumption were: 1) physical changes (benefits) not apparent, 2) inadequate salt-related health education, 3) hidden salt in food products, 4) inadequate food literacy (food labelling), 5) competitive pricing (affordability), 6) busy lifestyle, 7) low perceived susceptibility and 8) individual food taste preference and cooking habits. Interestingly, the research findings indicate that peer and family influences encouraged participants to adhere to a low-salt diet, but these influences could also discourage participants from sustaining such health behavioural changes. These results indicate that future health promotion strategies should focus on practical salt-reduction strategies such as salt alternatives and education on low-salt food choices and cooking methods, increasing awareness of salt-related health issues in the Chinese community and changing perceptions about salt reduction in the wider context so that salt reduction becomes a social norm. Further, because Asians share significant common dietary practices such as cooking with salt and condiments, the insights generated from this study may also be relevant and applicable to other Asian population groups.

Future studies are required to explore the factors influencing salt-related behaviours of Chinese Australians whose only language is Chinese, such as new migrants. This would not only increase the representation of this ethnic minority group in Australian primary healthcare research, but also ensure their needs and preferences for salt-related health education are addressed.

### Supplementary Information

**Additional file 1.** Guiding interview questions.

## Data Availability

All data generated or analyzed during this study are included in this published article.

## Abbreviations

GP: General practitioners
HBM: Health belief model
MSG: Monosodium glutamate
LGBTIQ +: Lesbian, gay, bisexual, transgender, intersex, queer or questioning, or another diverse gender identity
SD: Standard deviation
WHO: World Health Organization

## References

[1] World Health Organization. A global brief on hypertension: Silent killer, global publichealth crisis [Internet]. Geneva: WHO; 2013 [Available from: https://www.who.int/publications/i/item/a-global-brief-on-hypertension-silent-killer-global-public-health-crisis-world-health-day-2013.

[2] World Health Organization. Guideline: Sodium intake for adults and children [Internet]. Geneva: WHO; 2012 [Available from: https://www.who.int/publications/i/item/9789241504836. 23658998

[3] Land MA, Neal BC, Johnson C, Nowson CA, Margerison C, Petersen KS (2018). Salt consumption by Australian adults: a systematic review and meta-analysis. Med J Aust.

[4] Strohm D, Boeing H, Leschik-Bonnet E, Heseker H, Arens-Azevedo U, Bechthold A (2016). Salt intake in Germany, health consequences, and resulting recommendations for action. Ernahrungs Umschau.

[5] Tan M, He FJ, Wang C, MacGregor GA. Twenty-four-hour urinary sodium and potassium excretion in china: a systematic review and meta-analysis. J Am Heart Assoc. 2019;8(14):e012923–e. 10.1161/JAHA.119.012923 10.1161/JAHA.119.012923 PMC6662145 31295409

[6] Bhat S, Marklund M, Henry ME, Appel LJ, Croft KD, Neal B (2020). A systematic review of the sources of dietary salt around the world. Adv Nutr.

[7] De Cosmi V, Scaglioni S, Agostoni C (2017). Early taste experiences and later food choices. Nutrients.

[8] Fisher EB, Tang PY, Coufal MM, Liu Y, Jia W, Daaleman TP, Helton MR (2018). Peer support. Chronic illness care: Principles and practice.

[9] Goulding T, Lindberg R, Russell CG (2020). The affordability of a healthy and sustainable diet: an Australian case study. Nutr J.

[10] Song Z. Global Chinese Diaspora. In: Ratuva S, editor. The Palgrave handbook of ethnicity. Singapore: Palgrave Macmillan, Singapore; 2019. p. 1167–83.

[11] Wang Z, Chen Z, Zhang L, Wang X, Hao G, Zhang Z (2018). Status of hypertension in China. Circulation.

[12] Australian Bureau of Statistics. Australia's population by country of birth: Statistics on Australia's estimated resident population by country of birth. [Internet]. Canberra: Australian Bureau of Statistics; 2022 [Available from: https://www.abs.gov.au/statistics/people/population/australias-population-country-birth/2021.

[13] Australian Bureau of Statistics. ABS reveals insights into Australia’s Chinese population on Chinese new year [Internet]. Canberra: Australian Bureau of Statistics; 2018 [Available from: https://www.abs.gov.au/AUSSTATS/abs@.nsf/mediareleasesbytitle/D8CAE4F74B82D446CA258235000F2BDE.

[14] Lubbers M, Gijsberts M (2019). Changes in self-rated health right after immigration: a panel study of economic, social, cultural, and emotional explanations of self-rated health among immigrants in the Netherlands. Front Ecol Environ.

[15] Markides KS, Rote S (2018). The healthy immigrant effect and aging in the United States and other western countries. Gerontologist.

[16] Zou P (2019). Facilitators and barriers to healthy eating in aged Chinese Canadians with hypertension: a qualitative exploration. Nutrients.

[17] Saunders B, Sim J, Kingstone T, Baker S, Waterfield J, Bartlam B (2018). Saturation in qualitative research: exploring its conceptualization and operationalization. Qual Quant.

[18] Chan A, Chan SW-C, Kinsman L. Using the health belief model to understand the factors influencing the perceptions of people of Chinese ancestry about reducing salt consumption for hypertension prevention: A cross-sectional study. PLoS One. 2023;18(8):e0289867. 10.1371/journal.pone.0289867. 10.1371/journal.pone.0289867 PMC10431675 37585401

[19] Chan A, Chan SW-C, Khanam M, Kinsman L. Factors affecting reductions in dietary salt consumption in people of Chinese descent: An integrative review. J Adv Nurs. 2022;78(7):1919–37. 10.1111/jan.15237. 10.1111/jan.15237 PMC9323495 35384036

[20] Rosenstock IM, Strecher VJ, Becker MH (1988). Social learning theory and the Health Belief Model. Health Educ Q.

[21] Elo S, Kyngäs H (2008). The qualitative content analysis process. J Adv Nurs.

[22] Sandelowski M (1986). The problem of rigor in qualitative research. ANS Adv Nurs Sci.

[23] O'Brien BC, Harris IB, Beckman TJ, Reed DA, Cook DA (2014). Standards for reporting qualitative research: a synthesis of recommendations. Acad Med.

[24] Australian Bureau of Statistics. Average weekly earnings, Australia [Internet]. Canberra: Australian Bureau of Statistics; 2021 [Available from: https://www.abs.gov.au/statistics/labour/earnings-and-work-hours/average-weekly-earnings-australia/latest-release.

[25] Inguglia ES, Zhang Z, Tiwari BK, Kerry JP, Burgess CM. Salt reduction strategies in processed meat products – A review. Trends Cell Biol. 2017;59:70–8. 10.1016/j.tifs.2016.10.016

[26] Zhou D, Xi B, Zhao M, Wang L, Veeranki SP (2018). Uncontrolled hypertension increases risk of all-cause and cardiovascular disease mortality in US adults: the NHANES III Linked Mortality Study. Sci Rep.

[27] Zhang X, Hu X, Ma J, Zhang P, Li Y, Luo R (2020). Cluster randomised controlled trial of home cook intervention to reduce salt intake in China: a protocol study. BMJ Open.

[28] Leyvraz M, Mizéhoun-Adissoda C, Houinato D, Moussa Baldé N, Damasceno A, Viswanathan B (2018). Food consumption, knowledge, attitudes, and practices related to salt in urban areas in five sub-Saharan African countries. Nutrients.

[29] Nakadate M, Ishihara J, Iwasaki M, Kitamura K, Kato E, Tanaka J (2018). Effect of monitoring salt concentration of home-prepared dishes and using low-sodium seasonings on sodium intake reduction. Eur J Clin Nutr.

[30] Chudech S, Janmaimool P (2021). Effectiveness of warning graphic labels on cigarette packs in enhancing late-teenagers’ perceived fear of smoking-related harms in Bangkok, Thailand. J Public Health Res.

[31] Spicer JO, Coleman CG (2022). Creating effective infographics and visual abstracts to disseminate research and facilitate medical education on social media. Clin Infect Dis.

[32] van Kessel R, Wong BLH, Clemens T, Brand H (2022). Digital health literacy as a super determinant of health: more than simply the sum of its parts. Internet Interv.

[33] Michael V, You YX, Shahar S, Manaf ZA, Haron H, Shahrir SN (2021). Barriers, enablers, and perceptions on dietary salt reduction in the out-of-home sectors: a scoping review. Int J Environ Res Public Health.

[34] Mihardja L, Delima D, Massie RGA, Karyana M, Nugroho P, Yunir E (2018). Prevalence of kidney dysfunction in diabetes mellitus and associated risk factors among productive age Indonesian. J Diabetes Metab Disord.

[35] Crovetto M, Valladares M, Espinoza V, Mena F, Oñate G, Fernandez M, et al. Effect of healthy and unhealthy habits on obesity: a multicentric study. Nutrition. 2018;54:7–11. 10.1016/j.nut.2018.02.003 10.1016/j.nut.2018.02.003 29677480

[36] Mulders MDGH, Corneille O, Klein O. Label reading, numeracy and food & nutrition involvement. Appetite. 2018;128:214–22. 10.1016/j.appet.2018.06.003 10.1016/j.appet.2018.06.003 29886052

[37] Law QPS, Yau AHY, Chung JWY. Chinese adults' nutrition label literacy in Hong Kong: implications for nurses. Nurs Health Sci. 2019;21(2):171–7. 10.1111/nhs.12575 10.1111/nhs.12575 30345724

[38] Chen S, Shan LC, Tao W, Lu T, Regan Á, Han H (2020). A survey of Chinese consumers’ knowledge, beliefs and behavioural intentions regarding salt intake and salt reduction. Public Health Nutr.

[39] Ponzo V, Pellegrini M, Costelli P, Vázquez-Araújo L, Gayoso L, D’Eusebio C (2021). Strategies for reducing salt and sugar intakes in individuals at increased cardiometabolic risk. Nutrients.

