# Supplementary material for: “I use salt. However, I also use soy sauce, oyster sauce, sometimes chili sauce and….”: interviews with Australians of Chinese ancestry regarding reducing salt consumption for hypertension prevention
Source: BMC Nurs. 2023 Nov 6;22:414. doi: 10.1186/s12912-023-01576-3 (PMC10626782; doi:10.1186/s12912-023-01576-3)
Supplement: Supplementary file 1 — Additional file 1. Guiding interview questions. [file 12912_2023_1576_MOESM1_ESM.docx]

**ADDITIONAL FILE 1:** Guiding interview questions

Q1: Do you have high blood pressure or kidney disease? If any, how long have you been living with this chronic condition?

Q2: Understanding and knowledge of dietary salt on health:

- How much salt do you think you consume each day?
- Do you know the recommended daily salt intake by the World Health Organisation?
  - **If yes**, can you tell me where you have received this information from? Are you feeling confident in self-managing your salt intake?
  - **If no**, can you tell me whether you would like to receive the salt-related education and how you would like to receive the education?
- What do you know about the health risks associated with high dietary salt intake?

Q3: Perceptions of their own health risks in relation to the dietary behaviour (salt intake):

- Do you think your current dietary salt intake is a threat to your health?
  - - - **If you think it is a threat to your health,** how can we help you to improve your salt-related knowledge so you can make better food choices?
      - **If you don’t think it is a threat to your health,** how can we help you to change your mind/perception and do something about this for your health (perceived threat and action)?

Q4: Perceived health benefits of a low salt diet:

- What do you think the health benefits are to reducing the current level of salt intake to you and your family?
- Can you identify 3 most important factors that helped you to follow a low-salt diet?
- Can you tell me the reasons why **they are** important? Can you share your experience with me?

Q5: Perceived barriers to follow the recommended salt reduction strategies/interventions:

- Can you identify 3 barriers that prevent you from following a low-salt diet?
- Can you tell me the reasons why you perceive that they are your barriers? And/or why they are difficult to overcome? Can you share your experience with me?

Q6:

Option A: For participants with normal salt intake:

- how do you monitor the amount of salt in your diet? Can you share your experience with me?

OR

Option B: Preventive health action (for high salt intake participants only):

- What do you do to limit the amount of salt in your diet?
  - **If yes,** how exactly do you reduce your salt intake?
  - **If no,** can you tell me the reasons why you think salt reduction is not important?
- How do you measure what you consider to be enough salt in your cooked meal (or in cooking)?
- What alternative do you have to reduce salt in your diets?

Q7: Considering the health benefits and barriers of following a low salt diet:

- What are the most appropriate and effective salt reduction strategies in your situation? Eg. webinar
- Is there anything that can assist you to maintain your dietary behavioural changes in the long term?

Q8: Food labelling:

- Where do you find the information on what is contained in the food that you purchase from the supermarket?
- What do you look out for when you read a food label?
